# Clinical features and outcomes of COVID-19 in older adults: a systematic review and meta-analysis

**DOI:** 10.1186/s12877-021-02261-3

**Published:** 2021-05-19

**Authors:** Sunny Singhal, Pramod Kumar, Sumitabh Singh, Srishti Saha, Aparajit Ballav Dey

**Affiliations:** 1 Department of Geriatric Medicine, All India Institute of Medical Sciences, Ansari Nagar, Delhi, 110029 India; 2 Division of Endocrinology, Diabetes, Metabolism, and Nutrition, Mayo Clinic, Rochester, MN USA; 3 Division of Gastroenterology and Hepatology, Mayo Clinic, Rochester, MN USA

**Keywords:** Coronavirus, Mortality, Severe illness, Symptoms, Comorbidities

## Abstract

**Background:**

Few studies have focused on exploring the clinical characteristics and outcomes of COVID-19 in older patients. We conducted this systematic review and meta-analysis to have a better understanding of the clinical characteristics of older COVID-19 patients.

**Methods:**

A systematic search of PubMed and Scopus was performed from December 2019 to May 3rd, 2020. Observational studies including older adults (age ≥ 60 years) with COVID-19 infection and reporting clinical characteristics or outcome were included. Primary outcome was assessing weighted pooled prevalence (WPP) of severity and outcomes. Secondary outcomes were clinical features including comorbidities and need of respiratory support.

**Result:**

Forty-six studies with 13,624 older patients were included. Severe infection was seen in 51% (95% CI– 36-65%, *I*^*2*^–95%) patients while 22% (95% CI– 16-28%, *I*^*2*^–88%) were critically ill. Overall, 11% (95% CI– 5-21%, *I*^*2*^–98%) patients died. The common comorbidities were hypertension (48, 95% CI– 36-60% *I*^*2*^–92%), diabetes mellitus (22, 95% CI– 13-32%, *I*^*2*^–86%) and cardiovascular disease (19, 95% CI – 11-28%, *I*^*2*^*–*85%). Common symptoms were fever (83, 95% CI– 66-97%, *I*^*2*^–91%), cough (60, 95% CI– 50-70%, *I*^*2*^–71%) and dyspnoea (42, 95% CI– 19-67%, *I*^*2*^–94%). Overall, 84% (95% CI– 60-100%, *I*^*2*^–81%) required oxygen support and 21% (95% CI– 0-49%, *I*^*2*^–91%) required mechanical ventilation. Majority of studies had medium to high risk of bias and overall quality of evidence was low for all outcomes.

**Conclusion:**

Approximately half of older patients with COVID-19 have severe infection, one in five are critically ill and one in ten die. More high-quality evidence is needed to study outcomes in this vulnerable patient population and factors affecting these outcomes.

**Supplementary Information:**

The online version contains supplementary material available at 10.1186/s12877-021-02261-3.

## Background

A novel coronavirus, SARS-CoV-2 first emerged in December 2019, in Hubei province, China. From there, it spread rapidly across the world and was soon declared a Global Pandemic on March 11, 2020 [1]. Coronavirus disease 2019 (COVID-19) infection causes a respiratory illness, and is transmitted majorly through respiratory droplet and direct contact [2]. At the time of writing this review, more than 7 million confirmed cases and above 400,000 deaths have been reported worldwide [3]. These figures are expected to further increase as the pandemic is still evolving in many countries like India, Brazil, Russia and Africa, while a second wave is possible in countries showing decreasing trend [4].

Older adults have been found to be particularly susceptible to this infection. Early reports from China showed increased severity of illness and mortality among adults aged 60 years and above [5] and showed similar pattern in Europe with mortality reported to be as high as 10% in adults aged 70 years and above, compared to < 1% in young adults [6]. In comparison to younger adults, older patients have shown increased need for intensive care unit (ICU) admission and mechanical ventilation [7]. These findings are in- agreement with the clinical outcome of other respiratory viral infections like the Influenza and SARS (Severe Acute Respiratory Syndrome). Seasonal flu is also known to affect the older population and those with multiple co-morbidities more severely and associated with increased mortality, compared to younger adults [8, 9]. With SARS-CoV-2 infection, the mortality rate rises sharply in the age groups above 60 years. With more than 12% of population above 60 years of age, more than 800 million people around the world will fall in this vulnerable group [10]. Older patients are also known to present with atypical clinical features, and patients with respiratory infection may present with fatigue, anorexia and delirium, in the absence of fever and productive cough [11, 12]. This can lead to delayed diagnosis in these patients and further contribute to increase mortality.

Very few studies on COVID-19 have focus on the particularly vulnerable elderly patient group. In our review of literature, we came across a few case-series [13–15], and one review article reporting on the characteristics of COVID-19 infection in the older population [16]. This systematic review and meta-analysis was conducted with the aim to comprehensively describe the clinical presentation, co-morbidities, severity of disease and outcomes in older adults infected with COVID-19. By providing a complete description of COVID-19 illness among the older adults, we aim to improve the current understanding of this expanding pandemic and improve the care of the older patients with COVID-19.

## Methods

All procedures used in this systematic review and meta-analysis were consistent with the Preferred Reporting Items for Systematic Reviews and Meta-Analyses (PRISMA) guidelines [17]. The studies considered in this meta-analysis were observational studies that included older patients (≥ 60 years) with confirmed COVID-19 infection and reported comorbidities, clinical characteristics, severity of illness or outcome. Studies not reporting data for older patients separately, were excluded. Individual case reports or case series with < 10 old patients were also excluded.

A comprehensive search of PubMed and Scopus databases from December 2019 to May 3, 2020 was conducted. The search strategy was designed and conducted by the study’s investigators (S.S.2 and S.S.3). Controlled vocabulary supplemented with keywords was used to search for studies describing clinical characteristics, comorbidities and mortality of COVID-19 infection in older patients infected with COVID-19. Critical illness was either defined by the study’s definition or ICU admission. Severe illness included both severe only and critical patients. The actual strategy of listing all search terms used and how they are combined is available in the Supplement.

Two authors (S.S.1 and P.K.) independently reviewed the titles and abstracts of the identified studies, and those that did not answer the research question of interest were excluded. The remaining articles were reviewed to determine inclusion criteria fulfilment.

### Data abstraction

Data were independently abstracted to a predetermined data collection form by two investigators (S.S.1 and P.K.). Data collected for each study included study setting and design, month and year of publication, location, total number of patients, number of older adults, comorbidities, symptoms, laboratory findings, radiological findings, complications, respiratory support, severity of COVID-19 and mortality. Conflicts in data abstraction were resolved by consensus, referring to the original article.

### Methodological quality of included studies

Most of the studies included were case series. Hence, an appropriate tool was applied for assessment of risk of bias which was based on four domains i.e. selection, ascertainment, causality and reporting [18]. An assessment of overall quality of evidence was used to interpret the findings of the study. Data on most of our outcomes is expected to be of low quality, because the evidence arises from observational studies conducted in the midst of an ongoing pandemic. However, as the study question is clinically important, these studies were included in the systematic review and meta-analysis.

### Outcomes assessed

Our primary analysis focused on assessing weighted pooled prevalence (WPP) of severity of illness (severe, critically ill; defined as specified in included studies) and outcomes (death, discharge) in older patients with COVID-19 infection. Secondary outcomes were WPP of comorbidities, clinical features, laboratory and radiological findings and complications (acute kidney injury (AKI), acute respiratory distress syndrome (ARDS), acute liver injury and secondary infection). Need for respiratory support was assessed after excluding studies only including patients who were in the ICU or died from Covid-19, to avoid selection bias.

### Statistical analyses

We calculated WPP with corresponding 95% confidence intervals (CI) for each outcome. The inverse variance heterogeneity (IVhet) model of meta-analysis was used. The IVhet model is a modification of the fixed-effects models that accounts for between-study heterogeneity, while retaining individual weights of studies [19]. Freeman-Tukey double arcsine transformation was used in the calculation of WPP. Between study heterogeneity was assessed the *I*^*2*^ statistic,; *I*^*2*^ values greater than 50% suggest substantial heterogeneity [20]. Publication bias was assessed qualitatively by visual inspection of funnel plots and quantitatively by the Egger linear regression test (when more than 10 estimates were available in a single analysis) [21]. Subgroup analyses were done by study location (China vs outside China) for all variables (which had minimum 5 studies from both locations). Sensitivity analyses were done by excluding outlier studies. In addition, for analyses of outcomes (severity of illness and mortality), sensitivity analyses were performed by excluding studies which included only patients who were in the ICU or died. Statistical significance was set at *p*-value < 0.05. Calculations were performed and graphs constructed with MetaXL meta-analysis software (version 5.3; EpiGear International Pty Ltd).

## Results

**Fig. 1:**
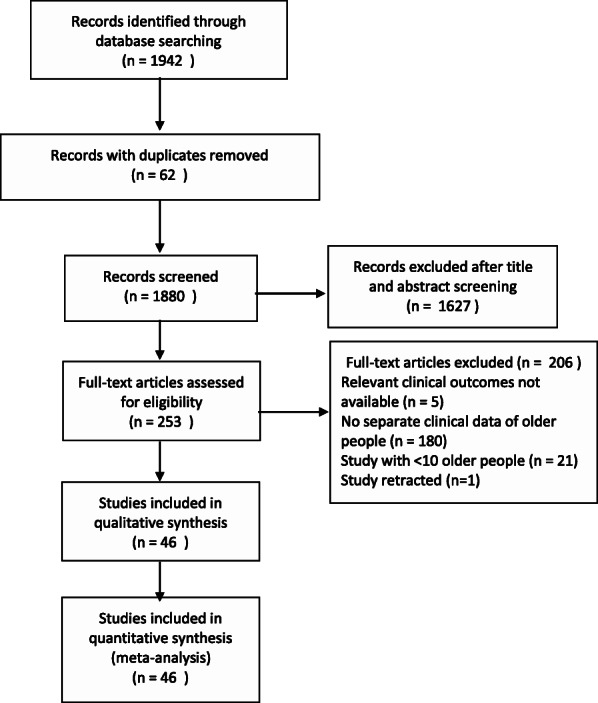
Study selection

Figure 1 shows the PRISMA flow diagram of the study selection process. A total of 1942 articles were identified in the initial search. After removing duplicates, 1880 were screened by titles and abstracts. Articles which were not available in English were translated with the help of Google Translate [22]. After screening by abstract and title, 253 articles were selected for full-text assessment. Out of these 253 articles, 206 articles were excluded after full-text review and finally 46 articles were included.

Out of 46 studies, most studies were limited to China (*n* = 26, 14 from Wuhan) followed by USA (*n* = 8), South Korea (*n* = 3), Italy (*n =* 3), one each from France, Spain and Iran. Two of them were multicentric studies which included patients from various American, Asian and European countries and one was from Diamond Princess Cruise ship. A total of 13,624 older patients were included. Supplementary Table S1 presents various characteristics of all the studies included in meta-analysis. The proportion of males in the studies ranged from 36 to 86%. Most studies were in the hospital setting (*n* = 38), while remaining included both inpatients and outpatients (*n* = 8). Most of the studies were of low (*n* = 14) or intermediate quality (*n* = 26) (Supplementary Table S1). 33 studies reported follow up; minimum follow up duration ranged from 0 to 35 days. The remaining 13 studies did not report duration of follow up. 15 studies didn’t have any minimum follow up period (0 days) i.e., the final date of enrolment was as same as the final date of outcome. As evident from the funnel plots (Supplementary Figs. S1-S8) and Egger’s regression analysis, there was publication bias for all variables.

### Severity of illness and outcome

**Fig. 2:**
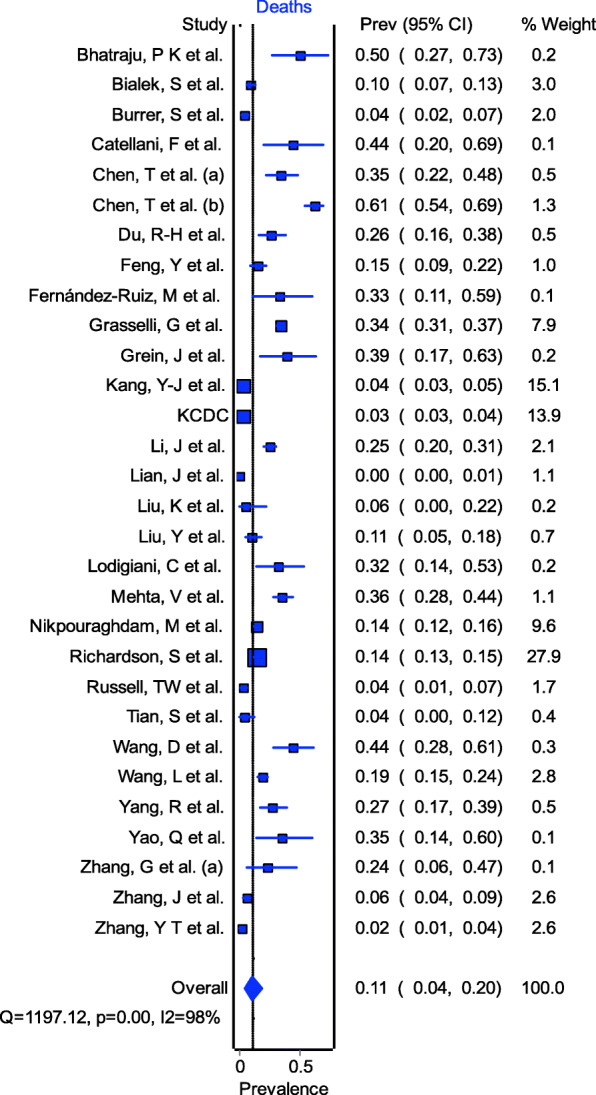
Weighted pooled prevalence of deaths among older patients with COVID-19

Table 1 provides details of severity of illness and outcomes in included studies. Overall, 50% (95% CI – 36-65%, *I*^*2*^–95%) of 2049 patients developed severe illness while 23% (95% CI – 16-31%, *I*^*2*^–88%) of 5280 progressed to critical illness (Supplementary Fig. S9 and S10). 43% (95% CI – 3-88%, *I*^*2*^–100%) of 6901 patients were discharged (Table 2). Overall, the WPP of mortality was 11% (95% CI – 4–20%, *I*^*2*^–98%) among all 12,060 older patients included in the study (Fig. 2).

**Table 1:** Severity of illness and outcomes of COVID-19 in included studies

Author name	n	Severe	Critical	Death	Discharged
Bhatraju, P K et al.	18	..	..	9	1
Bialek, S et al.	278	..	64	35	..
Burrer, S et al.^a^	238	..	..	10	..
Burrer, S et al.^a^	219	..	35	..	..
Catellani, F et al.	16	..	..	7	..
Chen, T (a)	55	48	24	19	36
Chen, T (b)	153	..	..	94	..
Chow, N et al.	715	..	232	..	..
Du, R-H et al.	65	..	..	17	..
Feng, Y et al.	118	45	25	18	87
Fernández-Ruiz, M et al.	15	3	..	5	6
Grasselli, G et al.	958	..	..	322	111
Grein, J et al.	18	..	..	7	..
Guan, W-J et al.	153	44	32	..	..
Kang, Y-J et al.	1825	..	..	67	..
KCDC	1679	..	..	58	..
Li, J et al.	259	135	..	65	194
Lian, J et al.	136	33	13	0	31
Liu, K et al.	18	4	..	1	17
Liu, Y et al.	85	..	..	9	76
Lodigiani, C et al.	22	..	4	7	11
Mehta, V et al.	138	..	..	49	..
Nikpouraghdam, M et al.	1164	..	..	160	..
Pereira, M R et al.	43	19	..	..	..
Richardson, S et al.^a^	3368	..	..	466	959
Richardson, S et al.^a^	2582	..	613	..	..
Russell, TW et al.	200	..	..	7	..
Tian, S et al.	48	20	..	2	..
Wang, D et al.	36		..	16	20
Wang, L et al.	339	239	80	65	91
Yang, R et al.	62	..	..	17	..
Yao, Q et al.	17	9	..	6	..
Yu, X et al.	107	21	..	..	..
Zhang, G et al. (a)	17	7	7	4	..
Zhang, G et al. (b)	62	24	..	..	..
Zhang, J et al.	315	246	67	19	..
Zhang, L et al.	19	11	11	..	..
Zhang, Y T et al.	312	114	36	6	..
Zhao, X-Y et al.	16	8	..	..	..

^a^Data for critical illness was not available for all patients; *KCDC* Korea Centers for Disease Control and Prevention, *ICU* Intensive care unit

**Table 2:** Weighted pooled prevalence (WPP) of comorbidities, clinical features, severity of illness and outcome in older patients with COVID-19

Variable	Number of studies	Number of patients	WPP	95% CI	***p***-value for Cochran’s Q	I^**2**^	Egger’s test* (***p***-value)
**Severity of illness**							
Severe	18	2049	0.50	0.36–0.65	0.00	95	< 0.001
Critical	14	5280	0.23	0.16–0.31	0.00	88	< 0.001
**Outcomes**							
Discharged	14	6901	0.43	0.03–0.88	0.00	100	< 0.001
Dead	30	12,060	0.11	0.04–0.20	0.00	98	< 0.001
**Comorbidities**							
≥ 1 comorbidity	12	1888	0.81	0.68–0.93	0.00	92	< 0.001
Hypertension	17	2245	0.48	0.36–0.60	0.00	92	< 0.001
Diabetes Mellitus	17	1804	0.22	0.13–0.32	0.00	86	0.004
Cardiovascular disease	13	1679	0.19	0.11–0.28	0.00	85	0.002
Hypothyroid	3	99	0.11	0.01–0.25	0.16	45	..
Neurological disease	9	871	0.09	0.06–0.13	0.02	55	..
Malignancy	12	1476	0.09	0.03–0.15	0.00	80	0.014
Chronic lung disease	14	1748	0.08	0.03–0.13	0.00	82	0.004
Cerebrovascular disease	9	814	0.08	0.06–0.11	0.23	24	..
Kidney disease	14	1591	0.05	0.01–0.09	0.00	77	0.066
Liver disease	11	1416	0.03	0.01–0.05	0.01	58	0.018
Autoimmune diseases	3	473	0.02	0.00–0.06	0.06	65	..
**Clinical Features**							
Fever	11	782	0.83	0.66–0.97	0.00	91	< 0.001
Cough	11	782	0.60	0.50–0.70	0.00	71	< 0.001
Dry cough	4	432	0.56	0.43–0.69	0.08	56	..
Sputum production	8	654	0.28	0.17–0.39	0.00	70	..
Dyspnoea	11	782	0.42	0.19–0.67	0.00	94	0.020
Fatigue	9	691	0.33	0.16–0.52	0.00	88	..
Anorexia	3	470	0.31	0.01–0.67	0.00	96	..
Chest discomfort	5	500	0.26	0.01–0.57	0.00	93	..
Diarrhoea	6	575	0.18	0.02–0.39	0.00	91	..
Myalgia	9	746	0.15	0.01–0.33	0.00	93	..
Abdominal pain	4	219	0.11	0.02–0.22	0.00	80	..
Sore Throat	5	639	0.10	0.00–0.25	0.00	94	..
Headache	7	714	0.09	0.00–0.24	0.00	94	..
Nausea-Vomiting	4	543	0.08	0.00–0.23	0.00	93	..
Gastrointestinal symptoms	3	169	0.15	0.00–0.79	0.00	94	..

### Baseline characteristics and comorbidities

On analysing comorbidities from various studies (Supplementary Table S2), 81% (95% CI – 68-93%, *I*^*2*^–92%) of older patients had at least one comorbidity (Table 2). Hypertension was the most common comorbidity (48, 95% CI – 36-60%, *I*^*2*^*–*92%) followed by diabetes mellitus (22, 95% CI – 13-32%, *I*^*2*^*–*86%) and cardiovascular disease (19, 95% CI – 11-28%, *I*^*2*^*–*85%) (Supplementary Fig. S11-S13). Two studies broadened their definitions of cardiovascular disease to include hypertension [23] or CVA [24] and hence were excluded from the analyses of these variables.

### Clinical features

Most common symptoms were fever (83, 95% CI – 66-97%, *I*^*2*^*–*91%), cough (60, 95% CI – 50-70%, *I*^*2*^*–*71%), dyspnoea (42, 95% CI – 19-67%, *I*^*2*^*–*94%) and fatigue (33, 95% CI- 16-52%, *I*^*2*^*–*88%) (Supplementary Fig. S14-S16). Among gastrointestinal symptoms, anorexia (31, 95% CI – 1-67%, *I*^*2*^*–*96%) followed by diarrhoea (18, 95% CI- 2-39%, *I*^*2*^*–*91%) were most common ones (Table 2). In some studies, presence or absence of gastrointestinal symptoms were reported without any additional details or specific information (Supplementary Table S3). These have reported as ‘gastrointestinal symptoms’ only. Wherever, specific information was available, it was reported and analysed separately.

### Laboratory and radiological findings

On analysing from various studies (Supplementary Table S4), lymphopenia (52, 95% CI – 24-81%, *I*^*2*^*–*94%) and leukopenia (20, 95% CI – 6-38%, *I*^*2*^*–*89%) were most commonly reported haematological findings while bilateral lung infiltrates (76, 95% CI – 44-100%, *I*^*2*^*–*97%) was the most common radiologic finding (Supplementary Table S7).

### Complications

Based on the few studies detailing complications in older patients (Supplementary Table S5), most common complications observed in these patients were secondary infection (34, 95% CI – 6-66%, *I*^*2*^*–*91%), AKI (26, 95% CI 0–65%, *I*^*2*^*–*98%) (Supplementary Table S7).

### Respiratory support

Very few studies have provided details of respiratory support in older patients (Supplementary Table S6). The WPP of requirement for oxygen support was 84% (95% CI – 60-100%, *I*^*2*^–81%). Overall, 21% (95% CI – 0-49%, *I*^*2*^–91%) required invasive mechanical ventilation (Supplementary Table S7). One study reported 68.4% mortality rate [13] while in the other one, out of two patients on mechanical ventilator, one died while the other one was in ICU for 21 days [25].

Sensitivity analysis was done by excluding outliers for severe illness, diabetes mellitus, chest discomfort, abdominal pain, sore throat, headache and lymphopenia. The pooled results did not change substantially. We further did analyses in which the studies which including only ICU patients [26, 27] or with clinical data for only dead patients [23, 24, 28–31] were excluded. No significant difference was seen in the pooled prevalence. We did a subgroup analysis according to the location of study (China vs. outside China) of the variables (Table 3). Death rate was similar in studies from China (15, 95% CI – 5-26%, *I*^*2*^–96%) and outside China (11, 95% CI – 3-20%, *I*^*2*^–98%) (*p* = 0.56) (Supplementary Fig. S17 and S18). Similarly, the WPP of comorbidities and clinical features were similar in both locations.

**Table 3:** Subgroup analysis of studies from China and outside China

Variable	China			Outside China			***p***-value for interaction
	Number of studies/patients	Prevalence (95% CI)	*p-*value (Cochran’s Q); I^2^	Number of studies/patients	Prevalence (95% CI)	*p-*value (Cochran’s Q); I^2^	
Death	16/2035	0.15 (0.05–0.26)	0.00; 96%	14/10025	0.11 (0.03–0.20)	0.00; 98%	0.56
≥1 comorbidity	6/410	0.68 (0.53–0.82)	0.00; 85%	6/1478	0.84 (0.72–0.95)	0.00; 91%	0.09
Hypertension	11/1387	0.42 (0.28–0.57)	0.00; 91%	6/858	0.57 (0.39–0.74);	0.00; 81%	0.20
Diabetes Mellitus	10/921	0.22 (0.10–0.36);	0.00; 90%	7/883	0.22 (0.12–0.33)	0.01; 65%	1.00
Cardiovascular disease	9/901	0.14 (0.08–0.20)	0.00; 72%	4/778	0.26 (0.11–0.43)	0.00; 79%	0.17
Chronic Lung disease	9/883	0.07 (0.04–0.11)	0.02; 56%	6/865	0.08 (0.00–0.26)	0.00; 91%	0.88
Malignancy	7/702	0.05 (0.03–0.07)	0.30; 17%	5/774	0.13 (0.01–0.28)	0.00; 78%	0.25
Kidney disease	8/720	0.04 (0.01–0.07)	0.06; 48%	6/871	0.06 (0.00–0.18)	0.00; 87%	0.68
Liver disease	6/582	0.02 (0.00–0.06)	0.02; 62%	5/834	0.04 (0.01–0.07)	0.09; 50%	0.85
Fever	6/583	0.85 (0.56–1.00)	0.00; 95%	5/199	0.77 (0.67–0.87)	0.08; 58%	0.42
Cough	6/583	0.57 (0.47–0.67)	0.03; 61%	5/199	0.71 (0.61–0.81)	0.11; 12.47%	0.05
Dyspnoea	6/583	0.36 (0.07–0.68)	0.00; 95%	5/199	0.63 (0.45–0.80)	0.00; 78%	0.13

The overall quality of evidence for all outcomes was low because of study design (observational studies only), lack of consistency of methodology, presence of publication bias and significant heterogeneity in all effect estimates.

## Discussion

In this systematic review and meta-analysis including 46 studies with 13,624 patients, we assessed clinical characteristics and outcomes of COVID-19 in older adults. Overall, half of the patients developed severe illness and 23% had critical illness or were admitted in ICU. Approximately one in ten patients died, and less than half were discharged from the hospital at the time of publication of the studies. Most studies were of low of intermediate quality, and there was significant heterogeneity and publication bias for all outcomes; overall quality of evidence was low.

The proportion of patients with severe illness amongst older patients is two-three times higher than reported in other meta-analyses (16.0–25.6%) [32–34]. The definition of severe or critical illness varied among studies. While most studies have used Chinese guidelines [35], some have used American Thoracic Society guidelines [36]. 11% of the older patients infected with coronavirus died during the hospital course. A marginal difference was observed when the subgroup analysis was done for patients in China (15%) and outside China (11%), which was not statistically significant. The published meta-analysis on COVID-19 has reported its mortality ranging from 3.1 to 5.5% [32–34, 37]. Hence, the fatality rate in our study is significantly higher, reflecting the poor resilience of older patients to COVID-19. However, most of the studies included in this systematic review were from hospital settings only. Hence, this may be higher than the true death rate of older adults in the general population.

As expected, comorbidities were quite common in older adults. Eight out of ten patients had at least one comorbidity with hypertension, diabetes, and cardiovascular disease being the most common. This finding is important as the presence of comorbidities such as hypertension or diabetes is considered as a predictor of adverse outcomes in these patents [32]. The WPP of hypertension and diabetes in our study was however similar to the ones reported in general population [38–42]. Further subgroup analysis showed similar WPP of comorbidities in patients irrespective of the location.

The most common symptoms seen in older patients were fever (83%), cough (60%) and dyspnoea (42%). Dry cough (56%) was more common than the productive cough (28%). However, while reporting cough, many studies didn’t specify the nature of cough, thus these results should be interpreted with caution. Gastrointestinal symptoms were also present with diarrhoea being the most common one. As compared to the other studies [33, 34, 37, 43, 44], fever and cough had similar WPP, however, dyspnoea was found to be more prevalent in older patients as compared to the younger ones. Similarly, though diarrhoea has been noted as the most common gastrointestinal symptom in other studies [33, 43] too, it has comparatively higher WPP in older population. It is important to note that gastrointestinal symptoms are common in older population, and patients and physicians should keep a low threshold of suspicion for COVID 19 even in the absence of typical symptoms. None of the included studies specified the proportion of patients presenting with respiratory and non-respiratory symptoms.

Since, most of the studies have not provided prevalence of abnormal laboratory findings in the cohort of older patients, we were able to extract data for only four variables. Lymphopenia (52%) followed by leukopenia (20%) were found to be quite common among the patients. Bilateral lung infiltrates (76%) was the most common radiological finding reported in older patients. These findings had a similar WPP to that reported in other age groups [33, 37, 43].

Most common complication seen in these patients is secondary infection (34%) followed by AKI (22%) and ARDS (20%). WPP of AKI and ARDS in others studies vary from 2.7 to 25.5% and 15.7 to 19.5% respectively [33, 37]. Of note, most of the patients (84%) required oxygen support and a significant number of patients (21%) required invasive mechanical ventilation. A few of them (4%) were also on non-invasive ventilation. Considering the high proportion of patients needing respiratory therapy, clinicians should keep a close watch on older patients with COVID-19 and keep a low threshold for hospitalization. Only 2 studies reported mortality among patients needing mechanical ventilation; future studies should explore this further.

Though there have been few meta-analyses describing the clinical characteristics and outcomes of COVID-19 patients [32–34, 37, 43, 44], this is the first one focussing on the older adults who are the most vulnerable patient cohort. We have reported on a comprehensive list of various comorbidities, clinical features, hospital course and outcomes. Unlike other systematic reviews and meta-analyses on COVID-19, we included studies from various centres and countries other than Wuhan, China. Since a large number of initial studies were from China, we also did subgroup analyses of studies from China and outside China. As the pandemic progressed, awareness of the disease, its manifestations and outcomes evolved. The subgroup analyses thus enable us to account for reporting bias and for regional differences in outcomes.

As this study comprised of data primarily from first wave, it is also important to view it within the context of subsequent waves of infection. As compared to the first wave, second wave infected patients were younger, though had similar clinical presentation but had lower mortality. However, the mean age of died patients was same or older as compared to the first wave [45–47]. This is interesting and needs to be studied further. Possible reasons may be increased awareness about the disease, new therapeutic options and new strains of the virus.

The study has several limitations. First, we found substantial heterogeneity between studies and significant publication bias for several variables. This may be due to the differences in study design, setting location, patient population, and sample size. Moreover, prevalence estimates are known to be limited by significant heterogeneity. Second, different lengths of follow-up and missing follow up information may bias results for several outcomes, particularly mortality. Some patients in the included studies were still in the hospital at the time of study publication. Lastly, some variables (clinical feature, e.g., nature of cough, severity of illness) were defined differently in included studies. Definition of older population varied in different studies which may affect outcomes. Also, none of the studies at the time measured frailty which can affect outcomes in COVID-19 [48]. Finally, most studies had a high or intermediate risk of bias, and overall quality of evidence was low.

## Conclusion

In this study of COVID-19 in the older population, we found a high proportion of patients with severe disease, critical illness and a high mortality. Further high-quality evidence is required, with a focus on older patients to improve our understanding and care of this disease.

## Supplementary Information

**Additional file 1. **Search Strategy for the systematic review and meta-analysis. **Supplementary References** (List of all eligible studies). **Supplementary Table S1** Characteristics of the studies included in meta-analysis. **Supplementary Table S2** Characteristics of studies with comorbidities. **Supplementary Table S3** Clinical features in included studies. **Supplementary Table S4** Laboratory and radiological findings in included studies. **Supplementary Table S5** Complications during hospital course. **Supplementary Table S6** Requirement for respiratory support in included studies. **Supplementary Table S7** Weighted pooled prevalence (WPP) of laboratory, radiological findings, complications and respiratory support in older patients with COVID-19. **Supplementary Fig. S1** Funnel plot depicting publication bias in meta-analyses for the prevalence of severe illness among older patients with COVID-19. **Supplementary Fig. S2** Funnel plot depicting publication bias in meta-analyses for the prevalence of critical illness among older patients with COVID-19. **Supplementary Fig. S3** Funnel plot depicting publication bias in meta-analyses for the prevalence of deaths among older patients with COVID-19. **Supplementary Fig. S4** Funnel plot depicting publication bias in meta-analyses for the prevalence of hypertension among older patients with COVID-19. **Supplementary Fig. S5** Funnel plot depicting publication bias in meta-analyses for the prevalence of diabetes mellitus among older patients with COVID-19. **Supplementary Fig. S6** Funnel plot depicting publication bias in meta-analyses for the prevalence of fever among older patients with COVID-19. **Supplementary Fig. S7** Funnel plot depicting publication bias in meta-analyses for the prevalence of cough among older patients with COVID-19. **Supplementary Fig. S8** Funnel plot depicting publication bias in meta-analyses for the prevalence of dyspnoea among older patients with COVID-19. **Supplementary Fig. S9** Weighted pooled prevalence of severe illness among older patients with COVID-19. **Supplementary Fig. S10** Weighted pooled prevalence of critical illness among older patients with COVID-19**. Supplementary Fig. S11** Weighted pooled prevalence of hypertension among older patients with COVID-19. **Supplementary Fig. S12** Weighted pooled prevalence of diabetes mellitus among older patients with COVID-19. **Supplementary Fig. S13** Weighted pooled prevalence of cardiovascular disease among older patients with COVID-19. **Supplementary Fig. S14** Weighted pooled prevalence of fever among older patients with COVID-19. **Supplementary Fig. S15** Weighted pooled prevalence of cough among older patients with COVID-19. **Supplementary Fig. S16** Weighted pooled prevalence of dyspnoea among older patients with COVID-19. **Supplementary Fig. S17** Weighted pooled prevalence of deaths among older patients with COVID-19 in China/ **Supplementary Fig. S18** Weighted pooled prevalence of deaths among older patients with COVID-19 outside China

## Data Availability

The datasets used and/or analysed during the current study are available from the corresponding author on reasonable request.

## Abbreviations

ARDS: Acute Respiratory Distress Syndrome
AKI: Acute Kidney Injury
IVhet: Inverse Variance heterogeneity
PRISMA: Preferred Reporting Items for Systematic Reviews and Meta-Analyses
SARS: Severe Acute Respiratory Syndrome
WPP: Weighted Pooled Prevalence
