# Supplementary material for: Clinical features and outcomes of COVID-19 in older adults: a systematic review and meta-analysis
Source: BMC Geriatr. 2021 May 19;21:321. doi: 10.1186/s12877-021-02261-3 (PMC8133052; doi:10.1186/s12877-021-02261-3)
Supplement: Supplementary file 1 — Additional file 1. Search Strategy for the systematic review and meta-analysis. Supplementary References (List of all eligible studies). Supplementary Table S1 Characteristics of the studies included in meta-analysis. Supplementary Table S2 Characteristics of studies with comorbidities. Supplementary Table S3 Clinical features in included studies. Supplementary Table S4 Laboratory and radiological findings in included studies. Supplementary Table S5 Complications during hospital course. Supplementary Table S6 Requirement for respiratory support in included studies. Supplementary Table S7 Weighted pooled prevalence (WPP) of laboratory, radiological findings, complications and respiratory support in older patients with COVID-19. Supplementary Fig. S1 Funnel plot depicting publication bias in meta-analyses for the prevalence of severe illness among older patients with COVID-19. Supplementary Fig. S2 Funnel plot depicting publication bias in meta-analyses for the prevalence of critical illness among older patients with COVID-19. Supplementary Fig. S3 Funnel plot depicting publication bias in meta-analyses for the prevalence of deaths among older patients with COVID-19. Supplementary Fig. S4 Funnel plot depicting publication bias in meta-analyses for the prevalence of hypertension among older patients with COVID-19. Supplementary Fig. S5 Funnel plot depicting publication bias in meta-analyses for the prevalence of diabetes mellitus among older patients with COVID-19. Supplementary Fig. S6 Funnel plot depicting publication bias in meta-analyses for the prevalence of fever among older patients with COVID-19. Supplementary Fig. S7 Funnel plot depicting publication bias in meta-analyses for the prevalence of cough among older patients with COVID-19. Supplementary Fig. S8 Funnel plot depicting publication bias in meta-analyses for the prevalence of dyspnoea among older patients with COVID-19. Supplementary Fig. S9 Weighted pooled prevalence of severe illness among older patie [file 12877_2021_2261_MOESM1_ESM.docx]

**Search Strategy for the systematic review and meta-analysis**

**PubMed**

| 1 | Search (("Corona virinae" OR "corona virus" OR coronaviridae OR coronavirus OR covid OR ncov) adj4 ("19" OR "2019" OR novel OR new)) OR (("Corona virinae" OR "corona virus" OR coronaviridae OR coronavirus OR covid OR ncov) and wuhan) OR "Corona virinae19" OR "Corona virinae2019" OR "corona virus19" OR "corona virus2019" OR Coronavirinae19 OR Coronavirinae2019 OR coronavirus19 OR coronavirus2019 OR COVID19 OR COVID2019 OR nCOV19 OR nCOV2019 OR "2019-nCOV" OR 2019nCOV OR "SARS Corona virus 2" OR "SARS Coronavirus 2" OR "SARS-COV-2" OR "Severe Acute Respiratory Syndrome Corona virus 2" OR "Severe Acute Respiratory Syndrome Coronavirus 2" Sort by: JournalName |
| --- | --- |
| 2 | Search "Symptoms" Sort by: JournalName |
| 3 | Search "clinical presentation" Sort by: JournalName |
| 4 | Search "clinical characteristics" Sort by: JournalName |
| 5 | Search "comorbidities" Sort by: JournalName |
| 6 | Search "comorbidity" Sort by: JournalName |
| 7 | Search intensive care units Sort by: JournalName |
| 8 | Search risk factors Sort by: JournalName |
| 9 | Search Hospitalization Sort by: JournalName |
| 10 | Search death Sort by: JournalName |
| 11 | Search mortality Sort by: JournalName |
| 12 | Search ((((((((("Symptoms") OR "clinical presentation") OR "clinical characteristics") OR "comorbidities") OR "comorbidity") OR intensive care units) OR risk factors) OR Hospitalization) OR death) OR mortality Sort by: JournalName |
| 13 | Search ((((("Corona virinae" OR "corona virus" OR coronaviridae OR coronavirus OR covid OR ncov) adj4 ("19" OR "2019" OR novel OR new)) OR (("Corona virinae" OR "corona virus" OR coronaviridae OR coronavirus OR covid OR ncov) and wuhan) OR "Corona virinae19" OR "Corona virinae2019" OR "corona virus19" OR "corona virus2019" OR Coronavirinae19 OR Coronavirinae2019 OR coronavirus19 OR coronavirus2019 OR COVID19 OR COVID2019 OR nCOV19 OR nCOV2019 OR "2019-nCOV" OR 2019nCOV OR "SARS Corona virus 2" OR "SARS Coronavirus 2" OR "SARS-COV-2" OR "Severe Acute Respiratory Syndrome Corona virus 2" OR "Severe Acute Respiratory Syndrome Coronavirus 2"))) AND (((((((((("Symptoms") OR "clinical presentation") OR "clinical characteristics") OR "comorbidities") OR "comorbidity") OR intensive care units) OR risk factors) OR hospitalization) OR death) OR mortality) Sort by: JournalName |
| 14 | Search ((((("Corona virinae" or "corona virus" or Coronavirinae or coronavirus or COVID or nCoV) adj4 ("19" or "2019" or novel or new)) or (("Corona virinae" or "corona virus" or Coronavirinae or coronavirus or COVID or nCoV) and wuhan) or "Corona virinae19" or "Corona virinae2019" or "corona virus19" or "corona virus2019" or Coronavirinae19 or Coronavirinae2019 or coronavirus19 or coronavirus2019 or COVID19 or COVID2019 or nCOV19 or nCOV2019 or "2019-nCOV" or 2019nCOV or "SARS Corona virus 2" or "SARS Coronavirus 2" or "SARS-COV-2" or "Severe Acute Respiratory Syndrome Corona virus 2" or "Severe Acute Respiratory Syndrome Coronavirus 2"))) AND (((((((((("Symptoms") OR "clinical presentation") OR "clinical characteristics") OR "comorbidities") OR "comorbidity") OR intensive care units) OR risk factors) OR Hospitalization) OR death) OR mortality) Sort by: JournalName |
| 15 | Search ((((("Corona virinae" OR "corona virus" OR coronaviridae OR coronavirus OR covid OR ncov) adj4 ("19" OR "2019" OR novel OR new)) OR (("Corona virinae" OR "corona virus" OR coronaviridae OR coronavirus OR covid OR ncov) and wuhan) OR "Corona virinae19" OR "Corona virinae2019" OR "corona virus19" OR "corona virus2019" OR Coronavirinae19 OR Coronavirinae2019 OR coronavirus19 OR coronavirus2019 OR COVID19 OR COVID2019 OR nCOV19 OR nCOV2019 OR "2019-nCOV" OR 2019nCOV OR "SARS Corona virus 2" OR "SARS Coronavirus 2" OR "SARS-COV-2" OR "Severe Acute Respiratory Syndrome Corona virus 2" OR "Severe Acute Respiratory Syndrome Coronavirus 2"))) AND (((((((((("Symptoms") OR "clinical presentation") OR "clinical characteristics") OR "comorbidities") OR "comorbidity") OR intensive care units) OR risk factors) OR hospitalization) OR death) OR mortality) Filters: Publication date from 2019/12/01 to 2020/05/03 |

**SCOPUS**

( TITLE-ABS-KEY ( ( ( ( "Corona virinae"  OR  "corona virus"  OR  coronavirinae  OR  coronavirus  OR  covid  OR  ncov )  W/4  ( "19"  OR  "2019"  OR  novel  OR  new ) )  OR  ( ( "Corona virinae"  OR  "corona virus"  OR  coronavirinae  OR  coronavirus  OR  covid  OR  ncov )  AND  wuhan )  OR  "Corona virinae19"  OR  "Corona virinae2019"  OR  "corona virus19" OR "coronavirus2019"  OR  coronavirinae19  OR  coronavirinae2019  OR  coronavirus19  OR  coronavirus2019  OR  covid19  OR  covid2019  OR  ncov19  OR  ncov2019  OR  "2019-nCOV"  OR  2019ncov  OR  "SARS Corona virus 2"  OR  "SARS Coronavirus 2"  OR  "SARS-COV-2"  OR  "Severe Acute Respiratory Syndrome Corona virus 2"  OR "Severe Acute Respiratory Syndrome Coronavirus 2" ) ) )  AND  ( symptom*  OR  clinical  AND presentation  OR  clinical  AND characteristics  OR  comorbidities  OR  comorb*  OR  comorbidity  OR  intensive  AND care  AND units  OR  intensive  AND care*  OR  risk  AND factor*  OR  hospitalization  OR  hospital*  OR  death  OR  mortality )  AND  ( LIMIT-TO ( PUBYEAR ,  2020 )  OR  LIMIT-TO ( PUBYEAR ,  2019 ) )

**Supplementary References**

List of all eligible studies:

1. Bhatraju PK, Ghassemieh BJ, Nichols M, et al. Covid-19 in Critically Ill Patients in the Seattle Region — Case Series. N Engl J Med. Published online March 30, 2020:NEJMoa2004500. doi:10.1056/NEJMoa2004500

2. Bialek S, Boundy E, Bowen V, et al. Severe Outcomes Among Patients with Coronavirus Disease 2019 (COVID-19) — United States, February 12–March 16, 2020. MMWR Morb Mortal Wkly Rep. 2020;69. doi:10.15585/mmwr.mm6912e2

3. Burrer S, Perio M, Hughes M, et al. Characteristics of Health Care Personnel with COVID-19 — United States, February 12–April 9, 2020. MMWR Morb Mortal Wkly Rep. 2020;69. doi:10.15585/mmwr.mm6915e6

4. Catellani F, Coscione A, D’Ambrosi R, Usai L, Roscitano C, Fiorentino G. Treatment of Proximal Femoral Fragility Fractures in Patients with COVID-19 During the SARS-CoV-2 Outbreak in Northern Italy: J Bone Jt Surg. Published online April 2020:1. doi:10.2106/JBJS.20.00617

5. Chen T, Dai Z, Mo P, et al. Clinical Characteristics and Outcomes of Older Patients with Coronavirus Disease 2019 (COVID-19) in Wuhan, China: A Single-Centered, Retrospective Study. Newman A, ed. J Gerontol Ser A. Published online April 11, 2020:glaa089. doi:10.1093/gerona/glaa089

6. Chen T, Wu D, Chen H, et al. Clinical characteristics of 113 deceased patients with coronavirus disease 2019: retrospective study. BMJ. Published online March 26, 2020:m1091. doi:10.1136/bmj.m1091

7. Chow N, Fleming-Dutra K, Gierke R, et al. Preliminary Estimates of the Prevalence of Selected Underlying Health Conditions Among Patients with Coronavirus Disease 2019 — United States, February 12–March 28, 2020. MMWR Morb Mortal Wkly Rep. 2020;69. doi:10.15585/mmwr.mm6913e2

8. Du R-H, Liang L-R, Yang C-Q, et al. Predictors of mortality for patients with COVID-19 pneumonia caused by SARS-CoV-2: a prospective cohort study. Eur Respir J. 2020;55(5):2000524. doi:10.1183/13993003.00524-2020

9. Feng Y, Ling Y, Bai T, et al. COVID-19 with Different Severity: A Multi-center Study of Clinical Features. Am J Respir Crit Care Med. Published online April 10, 2020:rccm.202002-0445OC. doi:10.1164/rccm.202002-0445OC

10. Fernández‐Ruiz M, Andrés A, Loinaz C, et al. COVID‐19 in solid organ transplant recipients: A single‐center case series from Spain. Am J Transplant. Published online May 10, 2020:ajt.15929. doi:10.1111/ajt.15929

11. Garg S, Kim L, Whitaker M, et al. Hospitalization Rates and Characteristics of Patients Hospitalized with Laboratory-Confirmed Coronavirus Disease 2019 — COVID-NET, 14 States, March 1–30, 2020. MMWR Morb Mortal Wkly Rep. 2020;69(15):458-464. doi:10.15585/mmwr.mm6915e3

12. Godaert L, Proye E, Demoustier-Tampere D, Coulibaly PS, Hequet F, Dramé M. Clinical characteristics of older patients: The experience of a geriatric short-stay unit dedicated to patients with COVID-19 in France. J Infect. Published online April 2020:S0163445320302176. doi:10.1016/j.jinf.2020.04.009

13. Grasselli G, Zangrillo A, Zanella A, et al. Baseline Characteristics and Outcomes of 1591 Patients Infected With SARS-CoV-2 Admitted to ICUs of the Lombardy Region, Italy. JAMA. 2020;323(16):1574. doi:10.1001/jama.2020.5394

14. Grein J, Ohmagari N, Shin D, et al. Compassionate Use of Remdesivir for Patients with Severe Covid-19. N Engl J Med. Published online April 10, 2020:NEJMoa2007016. doi:10.1056/NEJMoa2007016

15. Guan W, Ni Z, Hu Y, et al. Clinical Characteristics of Coronavirus Disease 2019 in China. N Engl J Med. 2020;382(18):1708-1720. doi:10.1056/NEJMoa2002032

16. Kang Y-J. Mortality Rate of Infection With COVID-19 in Korea From the Perspective of Underlying Disease. Disaster Med Public Health Prep. Published online March 31, 2020:1-3. doi:10.1017/dmp.2020.60

17. COVID-19 National Emergency Response Center, Epidemiology and Case Management Team, Korea Centers for Disease Control and Prevention. Coronavirus Disease-19: The First 7,755 Cases in the Republic of Korea. Osong Public Health Res Perspect. 2020;11(2):85-90. doi:10.24171/j.phrp.2020.11.2.05

18. Korean Society of Infectious Diseases and Korea Centers for Disease Control and Prevention. Analysis on 54 Mortality Cases of Coronavirus Disease 2019 in the Republic of Korea from January 19 to March 10, 2020. J Korean Med Sci. 2020;35(12):e132. doi:10.3346/jkms.2020.35.e132

19. Lechien JR, Chiesa‐Estomba CM, Place S, et al. Clinical and Epidemiological Characteristics of 1,420 European Patients with mild‐to‐moderate Coronavirus Disease 2019. J Intern Med. Published online April 30, 2020:joim.13089. doi:10.1111/joim.13089

20. Li J, Wang X, Chen J, Zhang H, Deng A. Association of Renin-Angiotensin System Inhibitors With Severity or Risk of Death in Patients With Hypertension Hospitalized for Coronavirus Disease 2019 (COVID-19) Infection in Wuhan, China. JAMA Cardiol. Published online April 23, 2020. doi:10.1001/jamacardio.2020.1624

21. Li T, Zhang Y, Gong C, et al. Prevalence of malnutrition and analysis of related factors in elderly patients with COVID-19 in Wuhan, China. Eur J Clin Nutr. Published online April 22, 2020. doi:10.1038/s41430-020-0642-3

22. Li X, Wang L, Yan S, et al. Clinical characteristics of 25 death cases with COVID-19: A retrospective review of medical records in a single medical center, Wuhan, China. Int J Infect Dis. 2020;94:128-132. doi:10.1016/j.ijid.2020.03.053

23. Lian J, Jin X, Hao S, et al. Analysis of Epidemiological and Clinical features in older patients with Corona Virus Disease 2019 (COVID-19) out of Wuhan. Clin Infect Dis. Published online March 25, 2020:ciaa242. doi:10.1093/cid/ciaa242

24. Lin L, Jiang X, Zhang Z, et al. Gastrointestinal symptoms of 95 cases with SARS-CoV-2 infection. Gut. 2020;69(6):997-1001. doi:10.1136/gutjnl-2020-321013

25. Liu K, Chen Y, Lin R, Han K. Clinical features of COVID-19 in elderly patients: A comparison with young and middle-aged patients. J Infect. 2020;80(6):e14-e18. doi:10.1016/j.jinf.2020.03.005

26. Liu Y, Mao B, Liang S, et al. Association between ages and clinical characteristics and outcomes of coronavirus disease 2019. Eur Respir J. Published online April 20, 2020:2001112. doi:10.1183/13993003.01112-2020

27. Lodigiani C, Iapichino G, Carenzo L, et al. Venous and arterial thromboembolic complications in COVID-19 patients admitted to an academic hospital in Milan, Italy. Thromb Res. 2020;191:9-14. doi:10.1016/j.thromres.2020.04.024

28. Mehta V, Goel S, Kabarriti R, et al. Case Fatality Rate of Cancer Patients with COVID-19 in a New York Hospital System. Cancer Discov. Published online May 1, 2020:CD-20-0516. doi:10.1158/2159-8290.CD-20-0516

29. Nikpouraghdam M, Jalali Farahani A, Alishiri G, et al. Epidemiological characteristics of coronavirus disease 2019 (COVID-19) patients in IRAN: A single center study. J Clin Virol. 2020;127:104378. doi:10.1016/j.jcv.2020.104378

30. Pereira MR, Mohan S, Cohen DJ, et al. COVID‐19 in solid organ transplant recipients: Initial report from the US epicenter. Am J Transplant. Published online May 10, 2020:ajt.15941. doi:10.1111/ajt.15941

31. Richardson S, Hirsch JS, Narasimhan M, et al. Presenting Characteristics, Comorbidities, and Outcomes Among 5700 Patients Hospitalized With COVID-19 in the New York City Area. JAMA. Published online April 22, 2020. doi:10.1001/jama.2020.6775

32. Russell TW, Hellewell J, Jarvis CI, et al. Estimating the infection and case fatality ratio for coronavirus disease (COVID-19) using age-adjusted data from the outbreak on the Diamond Princess cruise ship, February 2020. Eurosurveillance. 2020;25(12). doi:10.2807/1560-7917.ES.2020.25.12.2000256

33. Tian S, Hu N, Lou J, et al. Characteristics of COVID-19 infection in Beijing. J Infect. 2020;80(4):401-406. doi:10.1016/j.jinf.2020.02.018

34. Wang D, Yin Y, Hu C, et al. Clinical course and outcome of 107 patients infected with the novel coronavirus, SARS-CoV-2, discharged from two hospitals in Wuhan, China. Crit Care. 2020;24(1):188. doi:10.1186/s13054-020-02895-6

35. Wang L, He W, Yu X, et al. Coronavirus disease 2019 in elderly patients: Characteristics and prognostic factors based on 4-week follow-up. J Infect. Published online March 2020:S0163445320301468. doi:10.1016/j.jinf.2020.03.019

36. Wang W, Tang J, Wei F. Updated understanding of the outbreak of 2019 novel coronavirus (2019-nCoV) in Wuhan, China. J Med Virol. 2020;92(4):441-447. doi:10.1002/jmv.25689

37. Yang R, Gui X, Zhang Y, Xiong Y. The role of essential organ-based comorbidities in the prognosis of COVID-19 infection patients. Expert Rev Respir Med. Published online April 28, 2020:1-4. doi:10.1080/17476348.2020.1761791

38. Yao N, Wang S, Lian J, et al. Clinical characteristics and influencing factors of patients with novel coronavirus pneumonia combined with liver injury in Shaanxi region. Zhonghua Gan Zang Bing Za Zhi Zhonghua Ganzangbing Zazhi Chin J Hepatol. 2020;28:E003. doi:10.3760/cma.j.cn501113-20200226-00070

39. Yao Q, Wang P, Wang X, et al. Retrospective study of risk factors for severe SARS-Cov-2 infections in hospitalized adult patients. Pol Arch Intern Med. Published online April 24, 2020. doi:10.20452/pamw.15312

40. Yu X, Sun X, Cui P, et al. Epidemiological and clinical characteristics of 333 confirmed cases with coronavirus disease 2019 in Shanghai, China. Transbound Emerg Dis. Published online May 13, 2020:tbed.13604. doi:10.1111/tbed.13604

41. Zhang G, Zhang J, Wang B, Zhu X, Wang Q, Qiu S. Analysis of clinical characteristics and laboratory findings of 95 cases of 2019 novel coronavirus pneumonia in Wuhan, China: a retrospective analysis. Respir Res. 2020;21(1):74. doi:10.1186/s12931-020-01338-8

42. Zhang G, Hu C, Luo L, et al. Clinical features and short-term outcomes of 221 patients with COVID-19 in Wuhan, China. J Clin Virol. 2020;127:104364. doi:10.1016/j.jcv.2020.104364

43. Zhang J, Wang X, Jia X, et al. Risk factors for disease severity, unimprovement, and mortality in COVID-19 patients in Wuhan, China. Clin Microbiol Infect. Published online April 2020:S1198743X20302172. doi:10.1016/j.cmi.2020.04.012

44. Zhang L, Zhu F, Xie L, et al. Clinical characteristics of COVID-19-infected cancer patients: a retrospective case study in three hospitals within Wuhan, China. Ann Oncol. Published online March 2020:S0923753420363833. doi:10.1016/j.annonc.2020.03.296

45. Zhang YT, Deng AP, Hu T, et al. [Clinical outcomes of COVID-19 cases and influencing factors in Guangdong province]. Zhonghua Liu Xing Bing Xue Za Zhi Zhonghua Liuxingbingxue Zazhi. 2020;41(0):E057. doi:10.3760/cma.j.cn112338-20200318-00378

46. Zhao X-Y, Xu X-X, Yin H-S, et al. Clinical characteristics of patients with 2019 coronavirus disease in a non-Wuhan area of Hubei Province, China: a retrospective study. BMC Infect Dis. 2020;20(1):311. doi:10.1186/s12879-020-05010-w

Supplementary Table S1. Characteristics of the studies included in meta-analysis

| **Study name** | **Place** | **Patients characteristics** | **Type of study** | **Study duration** | **Minimum Length of follow up (days)** | **Cut-off age for definition of old people (years)** | **Number of old patient** | **Male** | **Age**  **(Median – Interquartile range)** | **Quality assessment** |
| --- | --- | --- | --- | --- | --- | --- | --- | --- | --- | --- |
| Bhatraju, P K et al.^1^ | USA | All patients admitted in ICU with COVID-19 | Retrospective case series | February 24, 2020 to March 9, 2020 | 14 | 60 | 18 | .. | .. | Medium |
| Bialek, S et al.^2^ | USA | All admitted patients with COVID-19 | Retrospective case series | February 12, 2020 to March 16, 2020 | None | 65 | 366 | .. | .. | Low |
| Burrer, S et al.^3^ | USA | All infected health care personnel with COVID-19 | Retrospective case series (surveillance data) | February 12, 2020 to April 9, 2020 | None | 65 | 238 | .. | .. | Low |
| Catellani, F et al.^4^ | Italy | All patients with hip fracture with fever or respiratory symptoms and diagnosed with COVID-19 | Prospective case series | N/A | 7 | 60 | 16 | 10 | 84.5 (82 - 88.8) | Low |
| Chen, T et al. (a)^5^ | China | All admitted patients with COVID-19 | Prospective Case series | January 1, 2020, to February 10, 2020 | 10 | 60 | 55 | 34 | 74(65-91) | High |
| Chen, T et al. (b)^6^ | China | All patients admitted with moderate to severe COVID-19 with a definite outcome | Retrospective case series | January 13, 2020 to February 28, 2020 | 16 | 60 | 153 | .. | .. | Medium |
| Chow, N et al.^7^ | USA | Patients aged >19 years with COVID-19 | Retrospective case series | February 12, 2020 to March 28, 2020 | None | 65 | 715 | .. | .. | Low |
| Du, R-H et al.^8^ | China | All admitted patients with COVID-19 | Prospective cohort study | December 25, 2019 to February 7, 2020 | .. | 65 | 65 | 10^#^ | 71 (66.5 - 77) | Medium |
| Feng, Y et al.^9^ | China | All admitted patients with COVID-19 | Prospective cohort study | January 1, 2020 to March 21, 2020 | 35 | 65 | 118 | 61 | .. | High |
| Fernández-Ruiz, M et al.^10^ | Spain | All solid organ transplant recipient patients admitted with COVID-19 | Prospective case series | March 5, 2020 to April4, 2020, | 14 | 60 | 15 | 12 | 72 (65 - 76) | Medium |
| Garg, S et al.^11^ | USA | All admitted patients with COVID-19 | Retrospective case series (surveillance data) | March 1, 2020 to March 30, 2020 | None | 65 | 73 | .. | .. | Medium |
| Godaert, L et al.^12^ | France | All older patients admitted with COVID-19 in a dedicated short stay geriatric center | Prospective case series | March 2020 first week | .. | 60 | 17 | 8 | .. | Medium |
| Grasselli, G et al.^13^ | Italy | All patients admitted in ICU with COVID-19 | Retrospective case series | February 20, 2020 and March 25, 2020 | 7 | 60 | 961 | 783 | .. | Medium |
| Grein, J et al.^14^ | Multicentric - USA, Japan, Canada, France, Austria, Italy, Germany, Netherland, Spain | All patients admitted with COVID-19 with oxygen saturation of 94% or less while they were breathing ambient air or who were receiving oxygen support and who do not have severe renal or hepatic dysfunction | Prospective cohort study | January 25, 2020 to March 30, 2020 | 28 | 60 | 18 | 6^#^ | 75 (72 - 78)## | Low |
| Guan, W-J et al.^15^ | China | All admitted and out-patients with COVID-19 | Retrospective case series | December 11, 2019, to January 31, 2020 | 8 | 65 | 153 | .. | .. | Medium |
| Kang, Y-J et al.^16^ | South Korea | All admitted patients with COVID-19 | Retrospective case series (surveillance data) | January 20, 2020 to March 16, 2020 | None | 60 | 1825 | .. | .. | Low |
| KCDC^17^ | South Korea | All admitted patients with COVID-19 | Retrospective case series (surveillance data) | January 20, 2020 to March 13, 2020 | None | 60 | 1679 | .. | .. | Low |
| KSID/KCDC^18^ | South Korea | All died patients with COVID-19 | Retrospective case series | January 19, 2020 to March 10, 2020 | .. | 70 | 34 | 20 | 78 (76.8–83.3) | Low |
| Lechien, J R et al.^19^ | Multicentric (France, Italy, Spain, Belgium and Switzerland) | All admitted patients with mild to moderate COVID-19 | Prospective case series | March 22, 2020 to April 10, 2020 | .. | 60 | 76 | 37 | .. | Medium |
| Li, J et al.^20^ | China | All admitted hypertensive patients with COVID-19 | Retrospective case series | January 15, 2020, to March 15, 2020 | None | 60 | 259 | .. | .. | Low |
| Li, T et al.^21^ | China | All admitted older patients with COVID-19 who do not have severe renal or hepatic dysfunction | Cross-sectional study | January to February 2020 | .. | 65 | 182 | 65 | .. | Medium |
| Li, X et al.^22^ | China | All died patients with COVID-19 | Retrospective case series | January 14, 2020 to Febuary 13, 2020. | .. | 60 | 19 | 9 | .. | Medium |
| Lian, J et al.^23^ | China | All admitted patients with COVID-19 | Retrospective case series | January 17, 2020 to February 12, 2020 | None | 60 | 136 | 58 | 68.3 +/- 7.3^**^ | Low |
| Lin, L et al.^24^ | China | All admitted patients with COVID-19 | Retrospective case series | January 17, 2020 to February 15, 2020 | None | 65 | 13 | .. | .. | Medium |
| Liu, K et al.^25^ | China | All admitted patients with COVID-19 | Retrospective case series | January 1, 2020 to February 15, 2020 | .. | 60 | 18 | 12 | 68.0 (65.3-69.8) | High |
| Liu, Y et al.^26^ | China | All admitted patients with COVID-19 diagnosed by fever clinics | Prospective case series | Upto March 7, 2020 | .. | 60 | 85 | .. | .. | Medium |
| Lodigiani, C et al.^27^ | Italy | All admitted patients with a thromboembolic event and COVID-19 | Retrospective cohort study | February 13, 2020 to April 10, 2020 | .. | 60 | 22 | 10 | 71 (67 - 76.5) | Medium |
| Mehta, V et al.^28^ | USA | All patients with cancer | Retrospective case series | March 18, 2020 to April 8, 2020 | 4 | 65 | 138 | 31^#^ | .. | Medium |
| Nikpouraghdam, M et al.^29^ | Iran | All admitted patients with COVID-19 | Retrospective case series | February 19, 2020 to April 15, 2020 | None | 60 | 1164 | .. | .. | Low |
| Pereira, M R et al.^30^ | USA | All admitted solid organ transplant recipient patient with COVID-19 | Prospective Case series | March 13, 2020 and April 3, 2020 | 20 (14-24)* | 60 | 43 | .. | .. | Medium |
| Richardson, S et al.^31^ | USA | All admitted patients with COVID-19 | Retrospective case series | March 1, 2020 to April 4, 2020 | None | 60 | 3368 | 753^#^ | .. | Medium |
| Russell, TW et al.^32^ | Diamond Princess Cruise ship | All patients on Diamond Princess Cruise ship with COVID-19 | Retrospective case series | January 25, 2020 to February 20, 2020 | None | 60 | 200 | .. | .. | Medium |
| Tian, S et al.^33^ | China | All admitted patients with COVID-19 | Retrospective case series | January 20, 2020 to February 10, 2020 | None | 65 | 48 | .. | .. | Low |
| Wang, D et al.^34^ | China | All discharged patients with COVID-19 | Retrospective case series | January 1, 2020 to February 10, 2020 | .. | 60 | 36 | .. | .. | High |
| Wang, L et al.^35^ | China | All admitted older patients with COVID-19 | Retrospective case series | January 1, 2020 to March 5, 2020 | 28 | 60 | 339 | 166 | 69 (65-76) | High |
| Wang, W et al.^36^ | China | All died patients with COVID-19 | Retrospective case series | December 1, 2019 to January 22, 2020 | .. | 60 | 15 | 12 | .. | Low |
| Yang, R et al.^37^ | China | All admitted patients with COVID-19 | Retrospective cohort study | January 11, 2020 to March 31, 2020 | 15 | 65 | 62 | .. | .. | Medium |
| Yao, N et al.^38^ | China | All admitted patients with COVID-19 | Retrospective case series | N/A | .. | 60 | 17 | .. | .. | Medium |
| Yao, Q et al.^39^ | China | All admitted patients with COVID-19 | Retrospective cohort study | January 30, 2020 to March 3, 2020 | 21 | 65 | 17 | .. | .. | High |
| Yu, X et al.^40^ | China | All admitted patients with COVID-19 | Retrospective case series | December 2019 to February 19, 2020 | None | 60 | 107 | .. | .. | Medium |
| Zhang, G et al. (a)^41^ | China | All admitted patients with COVID-19 who do not have chronic or severe underlying disease with Procalcitonin level <0.5 ng/ml | Retrospective case series | January 16, 2020 to February 25, 2020 | 7 | 60 | 17 | .. | .. | Medium |
| Zhang, G et al. (b)^42^ | China | All admitted patients with COVID-19 | Retrospective case series | January 2, 2020 to February 10, 2020 | 5 | 65 | 62 | .. | .. | Medium |
| Zhang, J et al.^43^ | China | All admitted patients with COVID-19 | Retrospective cohort study | January 11, 2020 to February 6, 2020 | 3 | 60 | 315 | .. | .. | Medium |
| Zhang, L et al.^44^ | China | Previously diagnosed cancer patients with COVID-19 | Retrospective cohort study | January 13, 2020 to February 26, 2020 | .. | 60 | 19 | 13 | 66 (65-71) | Medium |
| Zhang, Y T et al.^45^ | China | All admitted patients with COVID-19 | Retrospective case series | January 15, 2020 to march 4, 2020 | 20 | 60 | 312 | .. | .. | Medium |
| Zhao, X-Y et al.^46^ | China | All admitted patients with COVID-19 | Retrospective case series | January 16, 2020 to February 10, 2020 | None | 60 | 16 | .. | .. | Low |

*Median (interquartile range); **Mean ±SD;

#Data for number of males were available for limited patients.( Du, R-H et al – 20; Grein, J et al. – 7; Mehta, V et al. – 51; Richardson, S et al. – 1425)

KSID/KCDC - Korean Society of Infectious Diseases and Korea Centers for Disease Control and Prevention; KCDC - Korea Centers for Disease Control and Prevention

Supplementary Table S2. Characteristics of studies with comorbidities

| **Author name** | **n** | **≥1 comorbidity** | **DM** | **HTN** | **CVS disease*** | **CVA** | **Neurological disease**** | **Chronic lung disease** | **Kidney disease** | **Liver disease** | **Malignancy** | **Hypothyroid** | **Autoimmune disease** |
| --- | --- | --- | --- | --- | --- | --- | --- | --- | --- | --- | --- | --- | --- |
| Bhatraju, P K et al. | 18 | .. | .. | 16 | .. | .. | .. | .. | .. | .. | .. | .. | .. |
| Catellani, F et al. | 16 | 16 | 5 | 10 | 6 | 2 | 2 | .. | 2 | 1 | .. | 3 | .. |
| Chen, T et al. (a) | 55 | 37 | 12 | 21 | 11 | 8 | 8 | 7 | 3 | 2 | 5 | .. | .. |
| Chow, N et al. | 715 | 637 | .. | .. | .. | .. | .. | .. | .. | .. | .. | .. | .. |
| Du, R-H et al.^#^ | 20 | 16 | 20 | 13 | .. | .. | .. | .. | 2 | .. | 1 | .. | .. |
| Feng, Y et al. | 118 | 89 | 28 | 54 | 26 | 11 | 11 | 15 | 2 | .. | 8 | .. | .. |
| Garg, S et al. | ## | 67 | 20 | 45 | 32 | .. | 14 | 24 | 15 | 6 | .. | .. | 2 |
| Grasselli, G et al. | 635 | 481 | 135 | 363 | 175 | .. | .. | 33 | 24 | 18 | 69 | .. | .. |
| Grein, J et al.^#^ | 7 | 5 | 2 | .. | .. | .. | .. | 1 |  | .. | 1 | 2 | .. |
| KSID/KCDC | 34 | 31 | 11 | .. | .. | .. | .. | 6 | 3 | .. | 4 | .. | .. |
| Lechien, J R et al. | 76 | .. | 7 | 32 | 8 |  | 6 | 5 | 4 | 4 | 22 | 6 | 5 |
| Li, J et al. | 466 | .. | .. | 259 | .. | .. | .. | .. | .. | .. | .. | .. | .. |
| Li, T et al. | 182 | .. | 51 | 29 | 19 | 17 | 17 | 15 | .. | .. | .. | .. | .. |
| Li, X et al. | 19 | 19 | 6 | 14 | 6 | 2 | 2 | 2 | 3 | 1 | 2 | .. | .. |
| Lian, J et al. | 136 | 75 | 24 | 53 | 6 | .. | .. | 3 | 2 | 6 | 3 | .. | .. |
| Liu, K et al. | 18 | .. | 2 | 5 | 3 | .. | .. | .. | 0 | 1 | .. | .. | .. |
| Lodigiani, C et al. | 22 | .. | .. | .. | .. | .. | .. | .. | .. | .. | 6 | .. | .. |
| Mehta, V et al.^#^ | 51 | .. | 15 | 22 | 16 | 2 | 13 | 10 | 10 | 1 | .. | .. | .. |
| Wang, L et al. | 339 | .. | 54 | 138 | 53 | 21 | 21 | 21 | 13 | 2 | 15 | .. | 5 |
| Wang, W et al. | 15 | .. | 4 | 6 | 2 | 2 | 2 | 2 | 2 | 1 | 1 | .. | .. |
| Yang, R et al. | 62 | 39 | .. | .. | .. | .. | .. | .. | .. | .. | .. | .. | .. |
| Zhang, L et al. | 19 | .. | 3 | 3 | 3 | .. | .. | 1 | .. | .. | .. | .. | .. |

*Excluding Hypertension; **Including Cerebrovascular disease

# Though these studies included alive patients, however detailed clinical data for older patients was available only for dead patients;

## As data was not available for all variables, n varied for different variables (≥1 comorbidity - 71, HTN and Chronic lung disease - 62, DM - 64, CVS disease - 63, Neurological disease - 60, Kidney disease- 59, Liver disease – 56, Autoimmune disease - 58)

(DM- Diabetes Mellitus; HTN- Hypertension; CVS disease – Cardiovascular disease; CVA – Cerebrovascular diseases)

Supplementary Table S3. Clinical features in included studies

| **Author name** | **n** | **Cough** | **Dry cough** | **Sputum**  **production** | **Dyspnoea** | **Fever*** | **Sore throat** | **Diarrhoea** | **Abdominal pain** | **Fatigue** | **Myalgia** | **Headache** | **GI symptoms** | **Nausea/ Vomiting** | **Anorexia** | **Chest discomfort** |
| --- | --- | --- | --- | --- | --- | --- | --- | --- | --- | --- | --- | --- | --- | --- | --- | --- |
| Bhatraju, P K et al. | 18 | 15 | 10 | 6 | 15 | 13 | .. | .. | .. | .. | .. | .. | .. | .. | .. | .. |
| Chen, T et al. (a) | 55 | .. | 38 | .. | 33 | 52 | .. | 3 | 3 | 5 | 11 | 3 | .. | 2 | 5 | 36 |
| Du, R-H et al.^#^ | 20 | .. | 14 | 12 | 18 | 20 | .. | .. | .. | 13 | 7 | 5 | 8 | .. | .. | .. |
| Fernández-Ruiz, M et al. | 15 | 11 | .. | 1 | 11 | 14 | 1 | 3 | 1 | 3 | 3 | .. | .. | .. | .. | 1 |
| Garg, S et al. | 73 | 58 | .. |  | 54 | 62 | 11 | 21 | 3 |  | 19 | 7 | .. | 15 |  | .. |
| Godaert, L et al. | 17 | 12 | .. | 3 | 11 | 13 | .. | 6 | .. | 10 | 10 | .. | .. | .. | .. | .. |
| Lechien, J R et al. | 76 | 46 | .. | 13 | 34 | 52 | 30 | 36 | 18 | 35 | 37 | 39 |  | 20 | 48 | 7 |
| Lian, J et al. | 136 | 85 | .. | 49 | 17 | 74 | 17 | .. | .. | 24 | 20 | 8 | 11 | .. | .. | .. |
| Lin, L et al. | 13 | .. | .. | .. | .. | .. | .. | .. | .. | .. | .. | .. | 10 | .. | .. | .. |
| Liu, K et al. | 18 | 6 | .. | 6 | 2 | 14 | .. | .. | .. | 2 | .. | .. | .. | .. | .. | .. |
| Wang, L et al. | 339 | .. | 179 | 93 | 138 | 311 | 13 | 43 | .. | 135 | 16 | 12 | .. | 13 | 94 | 88 |
| Wang, W et al. | 15 | 8 | .. | 1 | 4 | 9 | .. | .. | .. | 5 | 1 | 1 | .. | .. | .. | 1 |

# Though these studies included alive patients, however detailed clinical data for older patients was available only for dead patients;

*Body temperature >38^0^C or 100.4^0^F

(GI symptoms – Gastrointestinal symptoms)

Supplementary Table S4. Laboratory and radiological findings in included studies

| **Author name** | **n** | **Leukopenia (<4000)** | **Leukocytosis (>10,000)** | **Lymphopenia (<1000)** | **Thrombocytopenia ( <1 lakh)** | **Single lobe lesion** | **Multiple lobe lesions** | **Bilateral Infiltrates** |
| --- | --- | --- | --- | --- | --- | --- | --- | --- |
| Chen, T et al. (a) | 55 | 20 | 10 | 45 | 9 | .. | .. | 54 |
| Feng, Y et al. | 118 | 9 | 24 | 70 | .. | 13 | .. | 105 |
| Fernández-Ruiz, M et al. | 15 | .. | .. | .. | .. | 5 | 3 | 4 |
| Godaert, L et al. | 17 | .. | .. | 13 | 7 | .. | .. | .. |
| Lian, J et al. | 136 | 38 | 9 | 42 | 7 | 15 | 59 | 57 |
| Liu, K et al. | 18 | 3 | 2 | .. | .. | 16 | 2 | .. |
| Liu, Y et al. | 85 | .. | .. | .. | .. | .. | .. | 76 |

Supplementary Table S5. Complications during hospital course

| **Author name** | **n** | **Secondary infection** | **Acute Kidney Injury** | **ARDS** | **Acute Liver Injury** |
| --- | --- | --- | --- | --- | --- |
| Fernández-Ruiz, M et al. | 15 | 2 | 2 | 5 |  |
| Godaert, L et al. | 17 | .. | 10 | .. | 8 |
| Lian, J et al. | 136 | .. | 3 | 23 | 10 |
| Liu, K et al. | 18 | 4 | .. | 4 | .. |
| Liu, Y et al. | 85 | 11 | .. | .. | .. |
| Richardson, S et al.* | 2578 | .. | 788 | .. | .. |
| Richardson, S et al.* | 2314 | .. | .. | .. | 40 |
| Wang, L et al. | 339 | 143 | 27 | 71 | 96 |
| Yao, N et al. | 17 | .. | .. | .. | 9 |

*Data for acute kidney injury and acute liver injury was not available for all patients. ARDS- Acute Respiratory Distress Syndrome

Supplementary Table S6. Requirement for respiratory support in included studies

| **Author name** | **n** | **Oxygen therapy** | **Non-invasive ventilation** | **Invasive mechanical ventilation** |
| --- | --- | --- | --- | --- |
| Catellani, F et al. | 16 | 14 | .. | .. |
| Chen, T (a) | 55 | 49 | .. | 24 |
| Fernández-Ruiz, M et al. | 15 | 6 | 3 | 2 |
| Lian, J et al. | 136 | .. | 3 | 6 |
| Liu, K et al. | 18 | 17 | 1 | 4 |
| Richardson, S et al. | 2582 | .. | .. | 558 |

KCDC - Korea Centers for Disease Control and Prevention

Supplementary Table S7. Weighted pooled prevalence (WPP) of laboratory, radiological findings, complications and respiratory support in older patients with COVID-19

| **Variable** | **Number of studies** | **Number of patients** | **WPP** | **95% CI** | **p-value for Cochran’s Q** | **I^2^** | **Egger’s test (p-value)** |
| --- | --- | --- | --- | --- | --- | --- | --- |
| **Laboratory findings** | | | | | | | |
| Leukocytosis | 4 | 327 | 0.13 | 0.05-0.23 | 0.01 | 75 | .. |
| Leukopenia | 4 | 327 | 0.20 | 0.06-0.38 | 0.00 | 89 | .. |
| Lymphopenia | 4 | 326 | 0.52 | 0.24-0.81 | 0.00 | 94 | .. |
| Thrombocytopenia | 3 | 208 | 0.10 | 0.00-0.30 | 0.00 | 88 | .. |
| **Radiological findings** | | | | | | | |
| Single lobe infiltrates | 4 | 287 | 0.16 | 0.00-0.46 | 0.00 | 94 | .. |
| Multiple lobe infiltrates | 3 | 169 | 0.38 | 0.09-0.69 | 0.01 | 80 | .. |
| Bilateral infiltrates | 5 | 409 | 0.76 | 0.44-1.00 | 0.00 | 97 | .. |
| **Complications** | | | | | | | |
| Secondary Infection | 4 | 457 | 0.34 | 0.06-0.66 | 0.00 | 91 | .. |
| Acute Kidney Injury | 5 | 3085 | 0.26 | 0.00-0.65 | 0.00 | 98 | .. |
| Acute Respiratory Distress Syndrome | 4 | 508 | 0.20 | 0.17-0.24 | 0.44 | 0 | .. |
| Acute Liver Injury | 5 | 2823 | 0.04 | 0.00-0.34 | 0.00 | 99 | .. |
| **Respiratory support** | | | | | | | |
| Oxygen Therapy | 4 | 104 | 0.84 | 0.60-1.00 | 0.00 | 81 | .. |
| Non-invasive ventilation | 3 | 169 | 0.04 | 0.00-0.16 | 0.04 | 69 | .. |
| Invasive ventilation | 5 | 2806 | 0.21 | 0.00-0.49 | 0.00 | 91 | .. |

Supplementary Figure S1. Funnel plot depicting publication bias in meta‐analyses for the prevalence of severe illness among older patients with COVID-19

Supplementary Figure S2. Funnel plot depicting publication bias in meta‐analyses for the prevalence of critical illness among older patients with COVID-19

Supplementary Figure S3. Funnel plot depicting publication bias in meta‐analyses for the prevalence of deaths among older patients with COVID-19

Supplementary Figure S4. Funnel plot depicting publication bias in meta‐analyses for the prevalence of hypertension among older patients with COVID-19

Supplementary Figure S5. Funnel plot depicting publication bias in meta‐analyses for the prevalence of diabetes mellitus among older patients with COVID-19

Supplementary Figure S6. Funnel plot depicting publication bias in meta‐analyses for the prevalence of fever among older patients with COVID-19

Supplementary Figure S7. Funnel plot depicting publication bias in meta‐analyses for the prevalence of cough among older patients with COVID-19

Supplementary Figure S8. Funnel plot depicting publication bias in meta‐analyses for the prevalence of dyspnoea among older patients with COVID-19

Supplementary Figure S9. Weighted pooled prevalence of severe illness among older patients with COVID-19

Supplementary Figure S10. Weighted pooled prevalence of critical illness among older patients with COVID-19

Supplementary Figure S11. Weighted pooled prevalence of hypertension among older patients with COVID-19

Supplementary Figure S12. Weighted pooled prevalence of diabetes mellitus among older patients with COVID-19

Supplementary Figure S13. Weighted pooled prevalence of cardiovascular disease among older patients with COVID-19

Supplementary Figure S14. Weighted pooled prevalence of fever among older patients with COVID-19

Supplementary Figure S15. Weighted pooled prevalence of cough among older patients with COVID-19

Supplementary Figure S16. Weighted pooled prevalence of dyspnoea among older patients with COVID-19

Supplementary Figure S17. Weighted pooled prevalence of deaths among older patients with COVID-19 in China

Supplementary Figure S18. Weighted pooled prevalence of deaths among older patients with COVID-19 outside China
